# Structure–property relationships in ABC-type polymer carriers: exploring drug loading and release behavior *via* dissipative particle dynamics simulation

**DOI:** 10.1039/d6ra02492k

**Published:** 2026-07-03

**Authors:** Zengwei Ma, Gaiqin Liu, Jianwei Wei

**Affiliations:** a College of Physics and New Energy, Chongqing University of Technology Chongqing 400054 China zwma@cqut.edu.cn redskywei@cqut.edu.cn

## Abstract

The topological architecture of polymer carriers is a critical determinant of their drug loading capacity and release characteristics. This study employed dissipative particle dynamics (DPD) simulations to systematically investigate the drug distribution and pH-responsive release behavior of three ABC-type polymer carriers with identical block compositions (where A is hydrophobic, B is pH-responsive, and C is hydrophilic) but distinct topologies: miktoarm star polymer, linear triblock copolymer, and star block copolymer. The simulation results revealed the fundamental differences in the drug distribution mechanism induced by the topological architecture of polymers. The miktoarm star polymer enables a unique “core–shell dual loading” mode, where drug molecules (doxorubicin, DOX) are simultaneously encapsulated within both the hydrophobic core and the pH-responsive intermediate layer. In contrast, within the micelles of linear and block-star copolymers, drug molecules are predominantly confined to the intermediate layer. Despite both the linear and star block copolymer systems exhibiting a “shell-loading” mode for drug distribution, the star block copolymers demonstrate a faster drug release under acidic conditions, whereas the linear block copolymers exhibit gradual drug release. Through the analysis of the interfacial electrostatic environment, we observed that a balanced ion distribution facilitates drug release in the star block copolymer system, whereas a counterion barrier arising from an overcharging effect impedes release in the linear block copolymer system. This study highlights the topological design as a robust strategy for precisely modulating both the spatial distribution and release behavior of therapeutic agents within nanocarriers.

## Abstract 2

DPD simulations are adopted to explore how topological architecture governs structure–property correlations of ABC-type polymer carriers, with distinct drug loading behaviors and release mechanisms observed among these delivery systems.

## Graphical abstract

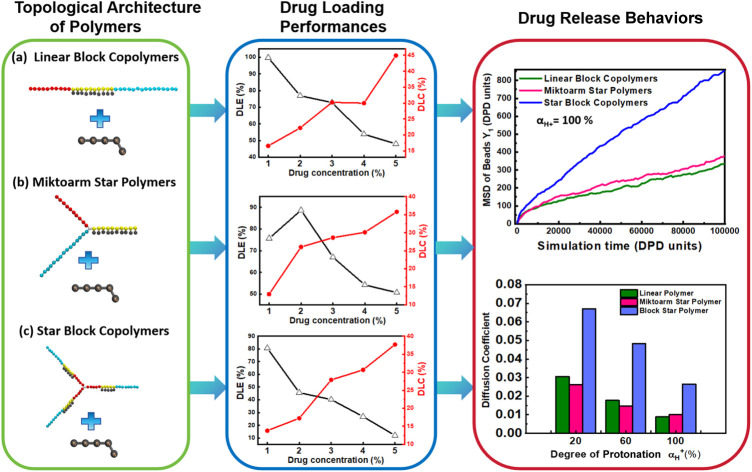

## 1. Introduction

Over the past few decades, micelles self-assembled from amphiphilic polymers have garnered extensive attention in the field of drug delivery.^1–3^ These nanocarriers typically feature a hydrophobic core for encapsulating hydrophobic drugs and a hydrophilic shell to maintain micellar stability in aqueous media.^4–6^ Given the increasing demand for sophisticated drug delivery systems in modern medicine, “smart” polymer carriers capable of responding to specific physiological environments, such as the micro-acidic milieu of tumor tissues, have emerged as a research hotspot.^7–13^ Among these, ABC three component polymers demonstrate substantial potential for constructing highly efficient drug delivery systems due to their structural versatility and tunability.^14–18^

The design of ABC-type polymers, composed of hydrophobic (A), environmentally responsive (B), and hydrophilic (C) blocks, enables the independent optimization of multiple properties, including drug encapsulation efficiency, environmental responsiveness, and biocompatibility.^19^ A critical factor governing their self-assembly behavior, micellar morphology, and overall functionality is the polymeric topological architecture.^20–23^ The spherical pH-responsive polymeric micelles with tunable aggregation-induced emission and controllable drug release were synthesized using the linear ABC triblock copolymer poly(ethylene glycol)–polyethylenimine–poly(ε caprolactone) (PEG–PEI–PCL), offering promise for simultaneous cell imaging and cancer therapy.^24^ With continuous advancements in synthesis technology, the research trend has progressively shifted from simple linear polymers to complex topological architectures for finer control over drug loading and release kinetics. Compared to their linear counterparts, star polymers exhibit multiple advantages, including smaller hydrodynamic diameter, enhanced drug-loading capacity, and reduced critical micelle concentration (CMC).^3,10^ Miktoarm star polymers, composed of at least two distinct polymer arms radiating from a central core, frequently demonstrate more complex self-assembly behaviors and superior properties.^1,5,17^ For instance, a miktoarm star polymer (MPEG)(PCL)(PPE) composed of a hydrophilic segment made of monomethoxy poly(ethylene glycol) (MPEG), a hydrophobic section of PCL, and thermosensitive polyphosphoester (PPE) chains forms thermal sensitivity micelles in an aqueous solution.^25^ These micelles exhibit spherical morphology at low temperatures but transform into nanorod structures at elevated temperatures. Additionally, a pH-responsive miktoarm star polymer (mPEO)_2_–P(His-*co*-BLG)–PLL has been employed for hydrophobic anticancer drug loading and controlled release, where PEO, P(His-*co*-BLG) and PLL refer to poly(ethylene oxide), poly(l-histidine-*co*-γ-benzyl-l-glutamate) and poly(l-lysine hydrochloride), respectively.^20^ The fabricated ‘stomatocytes’ suprapolymersomes display erythrocyte-like yet smaller architectures, enabling enhanced hemocompatibility and cellular uptake efficiency by overcoming hemorheological barriers in blood circulation.

Directly observing the internal drug distribution and dynamic release processes is experimentally challenging within these soft matter systems due to the large spatial and temporal scales involved. In this context, mesoscopic computer simulation methods, particularly dissipative particle dynamics (DPD), provide a powerful tool for exploring the phase behavior and dynamic processes of complex drug loading systems.^26–30^ By employing a coarse-grained model that preserves key chemical characteristics, the DPD method enables simulations on larger time and space scales, effectively bridging the gap left by experimental limitations in studying polymer self-assembly, morphological transitions, and drug release processes.^31–35^ For instance, Luo *et al.* utilized DPD method to simulate the drug loading and release characteristics of pH-sensitive amphiphilic linear polymers.^36^ Meanwhile, Nie *et al.* detailed the influence of different drug distributions on the pH-responsive release from linear ABC triblock copolymer micelles, proposing a “firework-like” structural transition model.^37^ Combined with experimental studies, DPD simulations were successfully used to investigate the drug-loading capacity and pH-responsive release behavior of A_2_(BC)_2_ miktoarm star polymers under both neutral and acidic conditions,^38^ with special emphasis on how polymer block ratios affect micelle properties. Yang *et al.* further extended the application of DPD simulations to star-like polymeric prodrug unimolecular micelles, systematically revealing their self-assembly mechanism, optimal preparation conditions, and superior dual pH/reduction-responsive drug release performance.^39^ These computational results are becoming indispensable for optimizing drug delivery systems (DDSs) and improving patient outcomes.

However, a systematic and direct comparative investigation into how ABC-type polymer topology fundamentally influences the spatial distribution of drug molecules within micelles and consequently dictates their release behavior remains relatively scarce, which hinders the rational design of advanced nanocarriers. Wu *et al.* employed DPD simulations to investigate the self-assembly, drug loading, and pH-responsive release of hertoarm star block polymers (PCL–PDEA-*b*-PPEGMA)_*n*_ and miktoarm (PCL)_*n*_(PDEA-*b*-PPEGMA)_*n*_.^40^ PDEA and PPEGMA represent poly[2-(diethylamino)ethyl methacrylate] and poly(ethylene glycol methyl ether methacrylate). They mainly reported that increasing the PDEA block length improved drug loading in miktoarm but reduced it in hertoarm systems due to the need for chain folding in the latter. Compared with Wu *et al.*'s work, the hydrophilic corona-forming block in their work was PPEGMA, whereas we adopted PEG as the hydrophilic block in our models. Our study mainly revealed the topology-dependent loading modes and the role of interfacial electrostatic environment in release kinetics for three ABC-type polymer carriers. Using DPD simulations, we comparatively investigated three ABC-type polymer carriers with identical chemical compositions and different topological structures: linear block, miktoarm star, and star block copolymers. We focused on two core questions: (1) how does polymer topology dictate the spatial distribution of drug molecules within the micelles at varying drug concentrations? (2) How does this topology-determined drug distribution pattern subsequently affect the pH-triggered release kinetics in acidic environments? Through this research, we aimed to elucidate the structure–property relationship, highlighting topology as a key design parameter governing drug delivery behavior.

## 2. Method and model details

### 2.1. DPD theory

The DPD method, initially proposed by Hoogerbrugge and Koelman^41^ and further developed by Groot and Warren,^32^ is a particle-based mesoscale simulation technique widely employed for investigating the phase behavior of complex fluids, such as polymer solutions, colloidal solutions, and biological systems.^34^ As a coarse-grained simulation approach, DPD enables the modeling of hydrodynamic phenomena across much larger temporal and spatial scales compared to atomistic simulations. Within this framework, each DPD bead—representing functional groups, molecules, or clusters of multiple atoms—adheres to Newton's equations of motion, providing a computationally efficient pathway to capture mesoscopic phenomena while retaining essential hydrodynamic interactions.

The force between each pair of beads is defined as the sum of five contributions: conservative force *F⃑*^C^_*ij*_, dissipative force *F⃑*^D^_*ij*_, random force *F⃑*^R^_*ij*_, bond force *F⃑*^S^_*ij*_, and electrostatic force *F⃑ij*^E^. The conservation force for non-bonded particles is defined by soft repulsion and taken as:1
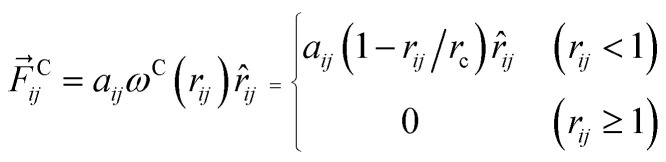
where *a*_*ij*_ is the interaction parameter between beads *i* and *j*, which reflects the chemical characteristics of interacting beads; *ω*^C^(*r*_*ij*_) is the weight function, and *r⃑*_*ij*_ = *r⃑*_*i*_ − *r⃑*_*j*_, *r*_*ij*_ = |*r⃑*_*ij*_|, *r̂*_*ij*_ = *r⃑*_*ij*_/*r*_*ij*_. *F⃑*^D^_*ij*_ corresponding to a frictional force depends on both the position and relative velocities of the beads and *F⃑*^R^_*ij*_ is a random interaction between bead *i* and its neighbor bead *j*. These two forces collectively act as an inherent thermostat that determines the system temperature, with their functional forms explicitly expressed through mathematical formulations:

2 *F⃑*^D^_*ij*_ = −*γω*^D^(*r*_*ij*_)(*ν⃑*_*ij*_·*r̂*_*ij*_)*r̂*_*ij*_ = −*γ*(1 − *r*_*ij*_/*r*_c_)^2^(*ν⃑*_*ij*_·*r̂*_*ij*_)*r̂*_*ij*_

3
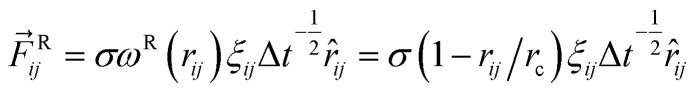
where *ν⃑*_*ij*_ = *ν⃑*_*i*_ − *ν⃑*_*j*_, *γ* and *σ* define the amplitude of the dissipative and the random forces, *ω*^D^(*r*_*ij*_) and *ω*^R^(*r*_*ij*_) are the weight functions. *ξ*_*ij*_ is a random number with zero mean and unit variance. According to the fluctuation-dissipative theorem, the following two relations are required:

4 *ω*^*D*^(*r*_*ij*_) = [*ω*^R^(*r*_*ij*_)]^2^, *σ*^2^ = 2*γk*_B_*T*

so that the system has a canonical equilibrium distribution. *F⃑*^S^_*ij*_ between bonding monomers is given by

5 *F⃑*^S^_*ij*_ = −*k*_s_(*r*_*ij*_ − *r*_0_)*r̂*_*ij*_

where *k*_s_ is the spring constant and *r*_0_ is the equilibrium bond length.

The electrostatic interactions are modeled using the Slater smearing charge distribution which means that bead charge spread out over a finite volume:

6 *ρ*(*r*) = *q* exp(−2*r*/*λ*)/π*λ*^3^

where *q* is the bead charge and *λ* is the decay length of charge. According to the method of González-Melchor *et al.*, the electrostatic force*F⃑*^E^_*ij*_ and electrostatic potential *U*^E^_*ij*_are expressed as^33,42^7
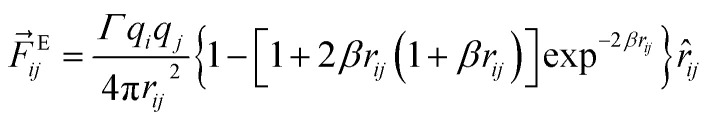
8
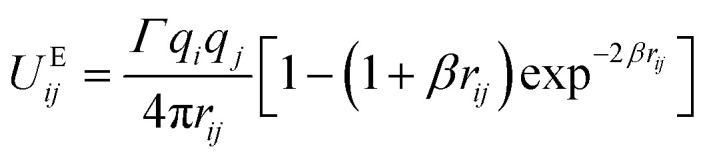
where *Γ* = *e*^2^/*k*_B_*Tε*_0_*ε*_r_*r*_c_, *e* is the elementary charge, *ε*_0_is the vacuum permittivity, *ε*_r_ is the relative permittivity of medium, and*β* = 5*r*_c_/8*λ*.

The interaction parameters *a*_*ij*_ can be calculated when the coarse-grained model of molecule has been established, which depends on the underlying atomistic interactions. Groot and Warren^32^ proposed the linear relationship between *a*_*ij*_ and Flory–Huggins *χ*_*ij*_ parameter when the number density of beads *ρ* in the system is set to 3:

9 *a*_*ij*_ = *a*_*ii*_ + 3.27*χ*_*ij*_

where the interaction parameter *a*_*ii*_ is set to 25 to ensure proper water compressibility when the bead density *ρ* = 3.

### 2.2. Model and parameters

**Fig. 1:**
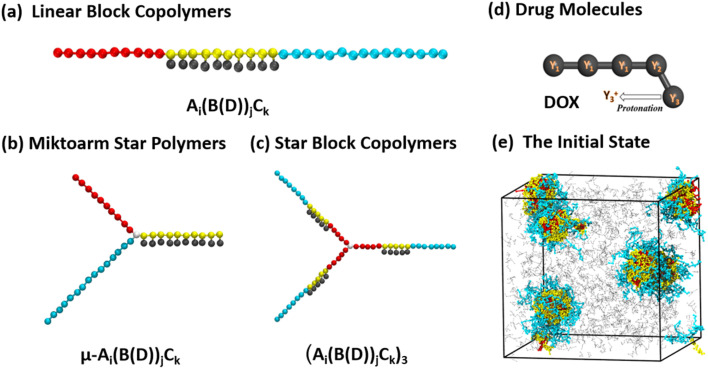
(a)–(c) Coarse-grained models of the three ABC-type polymer topologies; (d) coarse-grained model of the doxorubicin (DOX) molecule; (e) the initial state of multicomponent system with coarse-grained models for DPD simulations. Bead representations: A (red), B (yellow), C (cyan), D (gray), and DOX (black).

In this study, a pH-responsive drug delivery system was constructed, comprising ABC-type polymers, the drug molecule doxorubicin (DOX), and water molecules. The coarse-grained models of the polymers were derived from commonly used experimental structures. PCL, PDEA, and PEG were chosen as the hydrophobic, pH-responsive, and hydrophilic blocks respectively for the following reasons:^9^ (i) as a biodegradable and biocompatible polyester, PCL can form stable hydrophobic cores for drug encapsulation; (ii) PDEA undergoes pH-dependent protonation, reversible swelling and disassembly in the acidic tumor microenvironment, which endows it with excellent performance for pH-triggered drug release; (iii) PEG possesses low immunogenicity and stealth properties, and can stabilize micelles during blood circulation. As shown in Fig. 1a–c, the hydrophobic block PCL was represented by red bead A, the pH-responsive block PDEA was represented by two types of beads: yellow bead B for the main chain and gray bead D for the side chain, and the hydrophilic block PEG was represented by cyan bead C. The linear block copolymers were denoted as A_*i*_(B(D))_*j*_C_*k*_, the miktoarm star polymers were denoted as µ-A_*i*_(B(D))_*j*_C_*k*_, and the star block copolymers were denoted as (A_*i*_(B(D))_*j*_C_*k*_)_3_, where *i*, *j*, *k* represent the degrees of polymerization of the hydrophobic, pH-responsive, and hydrophilic segments respectively. Under acidic conditions, the D beads undergo protonation to form positively charged D^+^ beads. To maintain charge neutrality, counter-ions CI^−^ were incorporated into the system. DOX is represented by three types of beads (Y_1_, Y_2_, and Y_3_), with the tertiary amine group protonated in acidic environments to form Y_3_^+^ beads, as illustrated in Fig. 1d. Water molecules were represented by beads W. To investigate the drug loading mechanism, spherical micelles were self-assembled from various ABC-type polymers in aqueous solution (Fig. 1e). Drug molecules were initially homogeneously dispersed in the solution as the starting configuration for DPD simulations. The relationship between pH and the protonation degree of the pH-responsive arm was quantified using the Henderson–Hasselbalch equation:^43^10
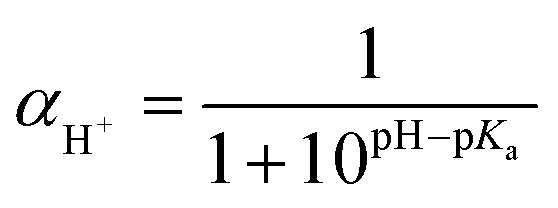
here *α*_H^+^_ represents the protonation degree, with p*K*_a_ = 6.9. It means that *α*_H^+^_ depends on the pH value of the solution. In a neutral environment (pH = 7.4), no beads D are protonated, and *α*_H^+^_ equals 0. As pH decreases, more beads D become protonated, and *α*_H^+^_ peaks at pH = 5.0, a condition that effectively mimics the tumor microenvironment.^7,8^

The interaction parameters *a*_*ij*_, listed in Table 1, were adopted from the work of Lin *et al.*, which demonstrated good agreement between DPD simulations and experimental findings for similar miktoarm star polymer systems.^38^ The parameters of the beads CI^−^ and Y_3_^+^ were the same as those of beads W and Y_3_, respectively. Simulations were performed in a cubic box of 40 × 40 × 40 *r*_e_^3^ with periodic boundary conditions. The bead number density was set to *ρ* = 3.0. The polymer concentration in solution was fixed at *f*_p_ = 5% and the drug concentration *f*_d_ was varied from 1% to 5%. The lengths of the hydrophobic, pH-responsive, and hydrophilic chains, were chosen as *i* = 10, *j* = 10, and *k* = 15, respectively. Based on the simulation parameters reported by Nie *et al.*,^37^ we adopted *i* = 10 for the hydrophobic block to form compact hydrophobic domains without macroscopic aggregation; *j* = 10 for the pH-responsive block to achieve sharp pH-triggered swelling and disassembly under tumor-mimetic acidic conditions; and *k* = 15 for the hydrophilic block to provide sufficient steric stabilization and prevent micellar fusion at neutral pH (7.4), matching the strategies employed in experimental drug delivery systems.^20^ The simulations were performed using the velocity-Verlet algorithm with a time step of Δ*t* = 0.05 and conducted under the canonical ensemble (NVT). Our simulations indicate that the simulation duration of 1.0 × 10^5^ steps is sufficient for the system to reach equilibrium, which is consistent with Guo *et al.*'s calculation.^44^ We defined the cutoff radius, the bead mass, and the temperature as the fundamental units of the simulation system, *i.e.*, *r*_c_ = *m* = *k*_B_*T* = 1.0. The friction coefficient *γ* and the noise amplitude *σ* were set to be 4.5 and 3.0, respectively. For the harmonic spring potential, the spring constant *k*_s_ was 4 and the equilibrium distance *r*_0_ was 0.^45^ The decay length *λ* = 0.67 and the smearing coefficient *β* = 0.929 are standard parameters, representing significant smeared charge distribution beyond the DPD bead.^45^ The permittivity coupling constant *Γ* was set to 13.87, corresponding to an aqueous environment. In the Ewald summation method, the electrostatic force was truncated at *r*cele = 3.0, real-space convergence parameter *α* = 0.975, and reciprocal vector range *n*_max_ = (5, 5, 5), empirically optimized for computational efficiency.^46^ All simulations were executed using the DL_MESO software package,^47^ and visualizations were generated with VMD.^48^

**Table 1:** Dissipative particle dynamics interaction parameters (*a*_*ij*_) between different beads

*a*_*ij*_	A	B	C	D	D^+^	O	Y_1_	Y_2_	Y_3_	W	CI^−^
A	25										
B	27	25									
C	37	28	25								
D	26	30	46	25							
D^+^	97	17	21	12	25						
O	28	35	53	26	27	25					
Y_1_	25	25	25	25	81	30	25				
Y_2_	25	25	25	25	87	30	25	25			
Y_3_	25	25	25	25	85	30	25	25	25		
W	53	35	26	37	11	82	44	27	24	25	
CI^−^	53	35	26	37	11	82	44	27	24	25	25

## 3. Results and discussion

Polymer topology exerts a profound influence on micellar self-assembly through the regulation of chain packing density, segmental mobility, and interfacial interaction parameters that collectively determine the microenvironmental properties essential for drug encapsulation and release.^49^ Compared to linear and conventional star architectures, miktoarm star polymers display asymmetric chain distribution from a central junction point, forming hierarchical nanospaces with well-defined hydrophobic and stimuli-responsive domains.^1,5^ This structural uniqueness enables simultaneous modulation of drug loading mechanisms and environmental responsiveness. The present study systematically compares three ABC-type polymer architectures to elucidate how topological variations affect micellar structure, drug distribution patterns, and delivery performance.

### 3.1. Influence of topology on micellar self-assembly and drug distribution

**Fig. 2:**
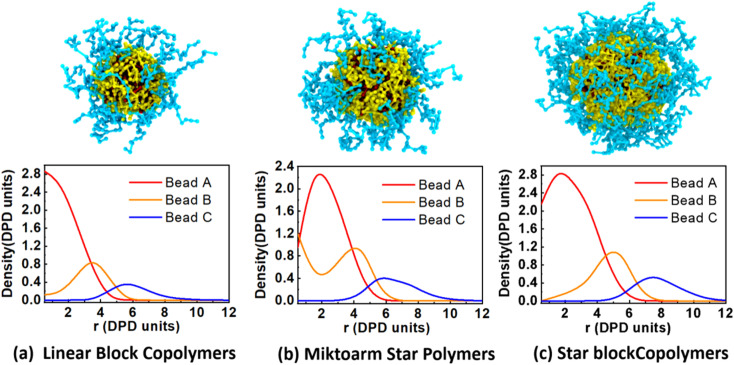
The morphologies and density profiles of A/B/C components for initial-state spherical micelles self-assembled from: (a) linear block copolymers; (b) miktoarm star polymers; (c) star block copolymers.

To elucidate the impact of polymer topology on drug loading mechanisms, we firstly investigated the self-assembly behavior under neutral physiological conditions (pH 7.4). All systems formed stable, spherical micelles at the initial state (Fig. 2), where the hydrophobic segments A constitute the core, the pH-responsive segments B form an intermediate layer, and the hydrophilic segments C compose the outer corona. Density distribution analyses revealed that the miktoarm star polymers formed micelles with a mixed core comprising both the hydrophobic segments A and the pH-responsive segments B (Fig. 2b). The hydrophobic A segments (red curve) and the pH-responsive B segments (yellow curve) exhibit significant overlap in the core region (*r* < 4 *r*_e_). Both A and B segments show substantial density at the micelle center, indicating that the pH-responsive B segments penetrate into and coexist with the hydrophobic A segments within the core region. This overlapping density distribution of A and B in the core region signifies a mixed core architecture, which originates from the geometric constraints of the miktoarm architecture: since all three arms emanate from a single central junction point, the conformational freedom of the B segments is restricted, preventing them from fully extending outward to form a well-separated intermediate layer. Instead, a portion of the B segments remains entrapped within the hydrophobic core domain, resulting in the mixed A/B core structure. Similar mixed-domain structures have been documented in systems such as (PCL)_3_–(PDEA-*b*-PPEGMA)_3_ miktoarm stars, where the restricted arm mobility induces partial penetration of the pH-responsive blocks into the hydrophobic core,^50^ a feature known to enhance both drug loading capacity and stimuli-responsiveness. In contrast, the linear and star block copolymers exhibited more pronounced core–shell stratification (Fig. 2a and c). The hydrophobic A segments (red curve) display a prominent density peak at the micelle center (*r* ≈ 0), which decays sharply as *r* increases, indicating that A segments are predominantly concentrated in the innermost region, forming a well-defined hydrophobic core. The pH-responsive B segments (yellow curve) show a density peak at an intermediate radial distance (*r* ≈ 3–5 *r*_e_), with minimal overlap with the A density peak, indicating that B segments form a distinct intermediate shell layer that is spatially separated from the hydrophobic core. This clear sequential ordering is the characteristic of a well-defined core–shell–corona architecture, where each block occupies a distinct spatial domain. Collectively, the miktoarm star topology promotes the formation of a mixed A/B core due to its constrained geometry, whereas linear and star block copolymers facilitate the formation of well-defined layered micelles.

**Fig. 3:**
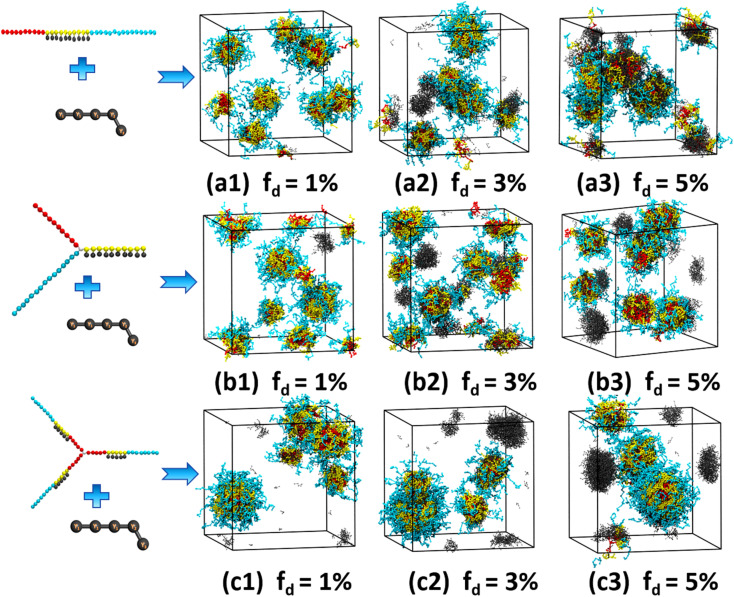
Equilibrium morphologies of drug-loaded polymeric micelles formed by the three polymer topologies at different drug concentrations *f*_d_: (a1–a3) linear block copolymers; (b1–b3) miktoarm star polymers; (c1–c3) star block copolymers.

This visual analysis of simulated equilibrium morphologies demonstrates that both polymer topology and drug concentration are pivotal determinants of the structure and stability of polymeric micelles. At low drug loading (*f*_d_ = 1%), all three investigated topologies successfully form stable, dispersed micelles. As the drug concentration increases to *f*_d_ = 3%, all three polymeric micelles exhibit a slight size increase, and drug precipitation is observed. At *f*_d_ = 5%, the micelles formed by the linear block copolymers show evidence of micellar fusion and irregular aggregation (Fig. 3a3), whereas miktoarm star polymers maintained spherical morphology (Fig. 3b3) even at this higher drug fraction. In contrast, the star block copolymers display the most pronounced drug expulsion (Fig. 3c3), indicating a reduced drug retention capacity. The superior stability of miktoarm systems aligns with previous findings by Lotocki *et al.*,^3^ who demonstrated that miktoarm star polymers resist dissociation under dilute conditions more effectively than their linear analogues. In summary, the morphology of drug-loaded polymeric micelles is highly sensitive to both polymer topology and drug loading fraction, with star polymers forming carriers of enhanced stability.

**Fig. 4:**
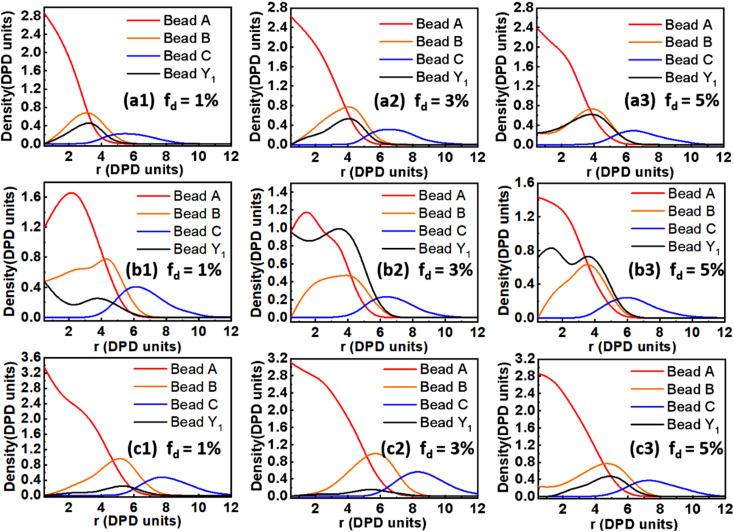
Density profiles of components (A, B, C, Y_1_) in drug-loaded polymeric micelles at different drug concentrations (*f*_d_ = 1%, 3%, and 5%): (a1)–(a3) linear block copolymers, (b1)–(b3) miktoarm star polymers, and (c1)–(c3) star block copolymers.

Quantitative density profile analysis (Fig. 4) further clarifies the topological influence on drug distribution. For both the linear and star block copolymers (depicted in the top and bottom rows, respectively), the primary density peak for the drug beads (Y_1_, black curve) indicates that the drug molecules are predominantly encapsulated within the micellar intermediate layer formed by pH-responsive segments, defined as a “shell-loading” mode. This observation aligns well with previous findings reported for linear ABC copolymers.^37^ In contrast, miktoarm star polymers display two distinct drug density peaks: one in the core region (*r* < 2 *r*_e_) overlapping with hydrophobic A-blocks, and another in the intermediate layer (*r* ≈ 2–4 *r*_e_) coinciding with B-block distribution, which has also been observed in a reported drug-loaded system formed by miktoarm star polymers (PCL)_2_(PDEA-*b*-PPEGMA)_2_.^38^ Our findings demonstrate that the miktoarm topology confers superior drug loading capacity through the “core–shell dual loading” mode, wherein hydrophobic drugs partition into both the hydrophobic core and the pH-responsive intermediate layer. This “core–shell dual loading” mode arises from the unique branching architecture, which reduces chain entanglement and creates additional binding sites within the micellar interior.^40,51^

**Fig. 5:**
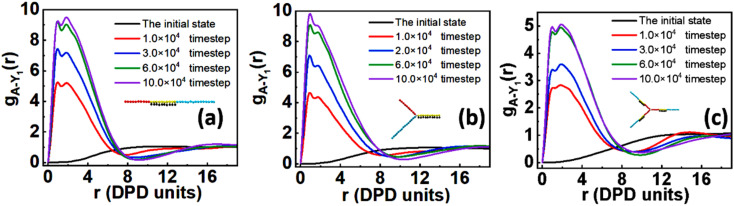
The radial distribution function *g*_A–Y1_(*r*) for the three polymeric micelles at different simulation time steps when *f*_d_ = 3%: (a) linear block copolymers, (b) miktoarm star polymers, (c) star block copolymers.

Fig. 5 illustrates the temporal evolution of the radial distribution function (RDF), *g*_A–Y1_(*r*), between component A and drug Y_1_ for the three polymeric micelle systems, revealing the kinetic characteristics and drug loading modes of the three drug delivery systems. The black curves *g*_A–Y1_(*r*) in Fig. 5 are all near to unity, indicating a predominantly disordered, dispersed state of the drug molecules. Over time, a sharp primary peak develops, reflecting the encapsulation of drug molecules into the micelles. The continuous increase in peak height signifies the gradual densification of drug molecules within the micellar core. For the linear block copolymer system, the primary *g*_A–Y1_(*r*) peak reaches a maximum of approximately 9.5 at *r* ≈ 2.0 *r*_e_. For the miktoarm star polymer, the highest peak appears at *r* ≈ 1.0 *r*_e_ with a value of about 10.0, indicating the strongest drug loading capacity in its core. In contrast, the star block copolymer behaves distinctly, showing a primary peak of only about 5.0 at *r* ≈ 2.5 *r*_e_, suggesting a “shell-loading” mode and a relatively weak loading capacity for drug molecules. These RDF results align perfectly with the drug distribution patterns observed in Fig. 4.

To quantitatively assess the drug loading performance, we calculated the drug loading efficiency (DLE) and drug loading content (DLC), which were calculated using the following equations:^52,53^11
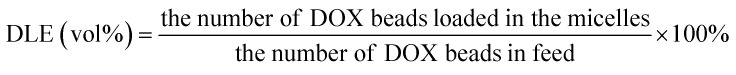
12
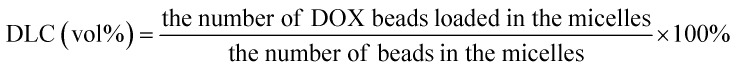


**Fig. 6:**
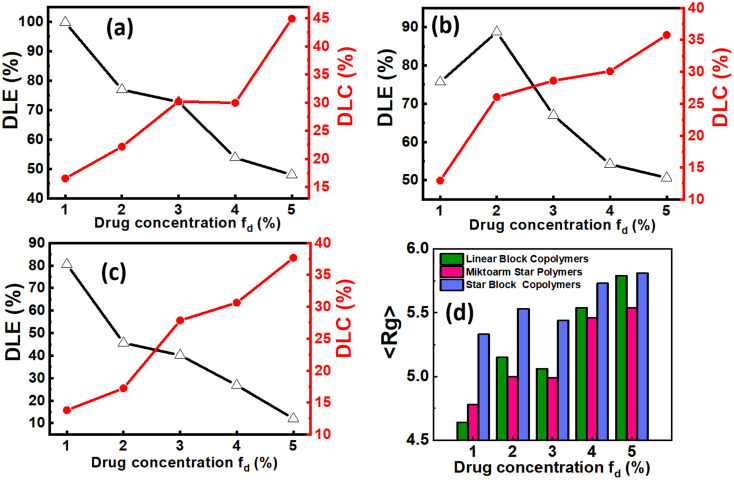
Drug loading efficiency (DLE) and drug loading content (DLC) for the three types of drug-loaded polymeric micelles as a function of drug concentration: (a) linear block copolymers; (b) miktoarm star polymers; (c) star block copolymers. (d) The average radius of gyration 〈*R*_g_〉 of three types of polymeric micelles at different drug concentrations.

Based on the drug loading performance data for the three polymeric micelles presented in Fig. 6, distinct and topology-dependent characteristics in drug encapsulation and delivery are evident. The DLE for the linear block copolymers exhibits nearly 100% at a low drug concentration (1%). This exceptional encapsulation capability stems from the flexible mobility of linear chain architecture, which promotes the formation of a stable core–shell micellar structure. This structural regularity enables efficient partitioning of hydrophobic drug molecules into the hydrophobic core, maximizing encapsulation at low concentrations. However, as the drug concentration increases to 5%, the DLE declines sharply to approximately 48%, indicating that excess drug molecules can no longer be effectively incorporated into micelles. Despite the DLE decreasing as the drug concentration increases, the DLC increases continuously from about 16% to 45%, which demonstrates that the absolute quantity of loaded drug still rises proportionally with the increased drug feeding. In contrast, the miktoarm star polymer displays a distinct DLE profile, peaking at approximately 90% efficiency at a 2% drug concentration. The DLE trend for the miktoarm star polymers aligns with the results observed in zwitterion-grafted dendrimers, which show a similar concentration-dependent loading.^53^ As the drug concentration increases further, the DLE gradually decreases to around 50%, while DLC shows a rise from 14% to 35%. The trends of DLE and DLC for the star block copolymers are similar to those of the linear block copolymers. The DLE decreases drastically as the drug concentration increases, dropping from 80% to only 12%. This marked reduction is mainly due to steric hindrance and intermolecular repulsion arising from its branched architecture. Nevertheless, its DLC still rises steadily from 12% to 38%, indicating that a higher absolute amount of drug can be loaded despite the relatively low loading efficiency.

The micellar size is characterized by the gyration radius *R*_g_:13
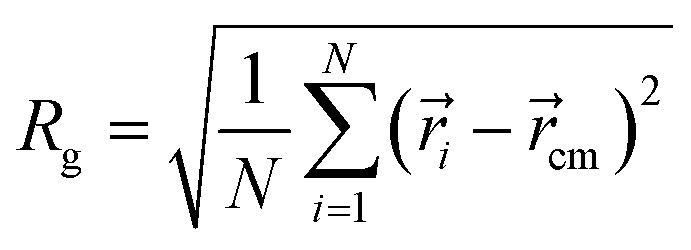
where *N* is the total number of beads in the aggregate; *r⃑*_*i*_ and *r⃑*_cm_ denote the position vectors of an individual bead and the center of mass of the aggregate, respectively. As illustrated in Fig. 6d, micellar sizes increase with the increase of the drug concentration across all systems, while the magnitude of change shows significant variation due to differences in topological structures. The stability of these micelles follows the order of star block copolymers, then miktoarm, and finally linear block copolymers, a hierarchy primarily attributable to differences in chain mobility. Star block polymers with their symmetric arm distribution easily form more compact and rigid structures that resist swelling.^49^ Miktoarm star polymers, despite their branched architecture, exhibit greater flexibility than star block copolymers due to asymmetric arm distribution, leading to moderate swelling. Linear block copolymers, lacking structural constraints, can undergo significant chain extension and micellar fusion.^43^ The minimal size change of star block polymers may improve circulation stability but limits drug loading capacity. In contrast, the moderate swelling of miktoarm systems balances stability and loading efficiency, potentially enhancing tumor accumulation through the enhanced permeability and retention (EPR) effect.^17,28^ Based on a comprehensive analysis of the drug-loading performance and micellar size variation with drug concentration as presented in Fig. 6, we propose the following structure–property relationships. The linear block copolymers achieve the highest drug loading content (DLC) owing to the flexible chain mobility, which permits significant core expansion to accommodate more drug molecules. In contrast, the star block copolymers exhibit the best dimensional stability, originating from strong topological constraints imposed by the branched architecture. Compared to the two situations mentioned above, the miktoarm star polymers effectively balance drug-loading capacity and micellar stability.

### 3.2. pH-triggered drug release behavior

**Fig. 7:**
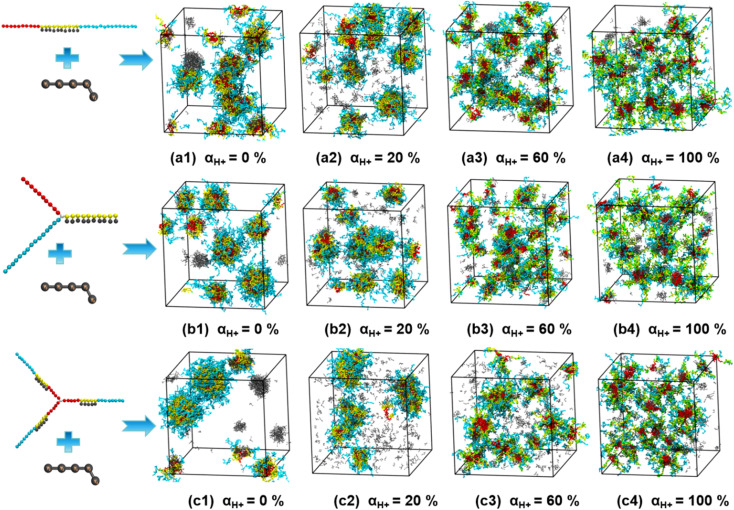
Morphologies of drug-loaded micelles formed by the three polymer topologies at different degrees of protonation *α*_H^+^_: (a1)–(a4) linear block copolymers, (b1)–(b4) miktoarm star polymers, and (c1)–(c4) star block copolymers.

The distinct drug distribution patterns, inherently determined by the polymer topology, directly and profoundly influence the drug release behaviors of the micelles in acidic environments. When ambient pH decreases, the side chains (D beads) of the pH-responsive B-block (PDEA) undergo protonation, acquiring a positive charge (D^+^). The electrostatic repulsion among these charged segments induces significant stretching of the pH-responsive B-block chains, leading to substantial structural reorganization of the micelles and consequently triggering drug release.^8,54^ In this section, we set *f*_d_ = 2% to clearly observe the drug release behavior. The morphological transformations of drug-loaded micelles formed by distinct polymer topological structures under varying protonation degrees *α*_H^+^_ are presented in Fig. 7. We have systematically investigated pH effects on micellar morphologies by setting *α*_H^+^_ = 20%, 60%, and 100%, corresponding to decreasing pH values. At *α*_H^+^_ = 20% (Fig. 7a2–c2), micelles formed by linear block copolymers and miktoarm star polymers exhibit size enlargement due to pH-responsive chain extension, while micelles formed by the star block copolymers break up into more small spherical micelles under the weak electrostatic interaction. As *α*_H^+^_ increases to 60% and 100%, drug-loaded micelles transform into numerous small aggregates under the strong electrostatic interaction. Fig. 7 reveals that drug molecules can be effectively released into solution during the morphological transition, while a portion of drug molecules remains in hydrophobic aggregate cores due to drug–water hydrophobic interactions. In summary, as the degree of protonation increases, micelles derived from all three polymer topologies progressively transform from well-defined spherical structures into smaller aggregates, demonstrating a consistent and effective mechanism for pH-triggered drug release.

**Fig. 8:**
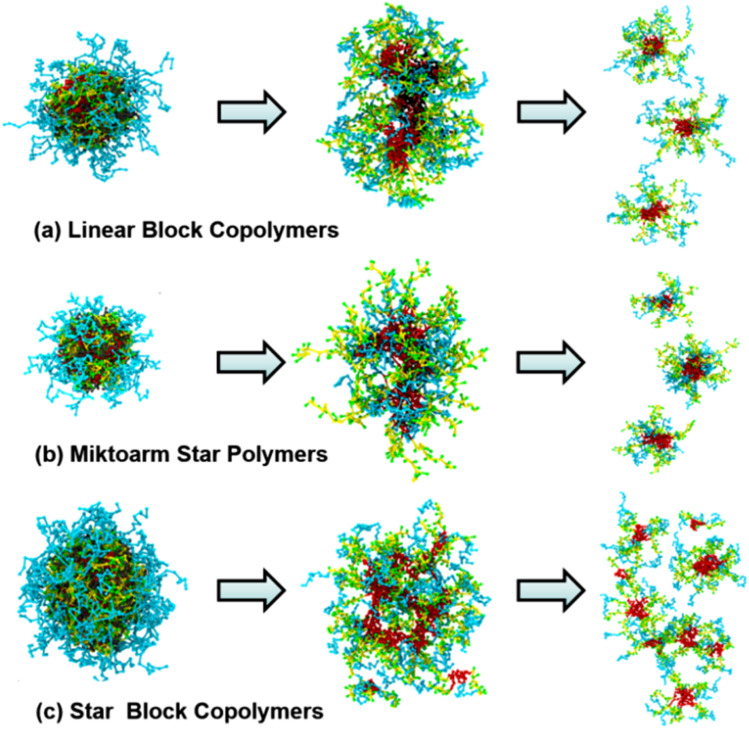
Morphological transformation for the three types of drug-loaded micelles (*f*_d_ = 2%) in an acidic environment (*α*_H^+^_ = 100%, pH = 5.0): (a) linear block copolymers; (b) miktoarm star polymers; (c) star block copolymers. The sequence from left to right shows the initial state at timestep = 0, the swollen state at timestep = 1000, and the disintegrated state in equilibrium.

Fig. 8 illustrates the morphological transformation processes for the three types of drug-loaded micelles under acidic conditions (pH = 5.0, *α*_H^+^_ = 100%), facilitating the understanding of their disassembly mechanisms. All micelles exhibit rapid swelling in Fig. 8, with their B-blocks extending outwards to form a distinctive ‘firework-like’ structure, agreeing well with a previous simulation result.^18^ This swift structural transition generates numerous transient channels, helping drug molecules diffuse into solution. Detailed comparison of the ‘firework-like’ structures at timestep = 1000 (Fig. 8a–c) reveals topology-dependent responsive behaviors. For the linear block copolymers in Fig. 8a, the B-blocks can just extend to the border of the hydrophilic shell at timestep = 1000, while the B-blocks can obviously extend to the outside of the hydrophilic layer for the miktoarm star polymers in Fig. 8b. The B-blocks remain confined within the hydrophilic layer for the star block copolymers in Fig. 8c. The difference in polymer topology is the fundamental cause for the variation in the above pH-responsive behavior.

**Fig. 9:**
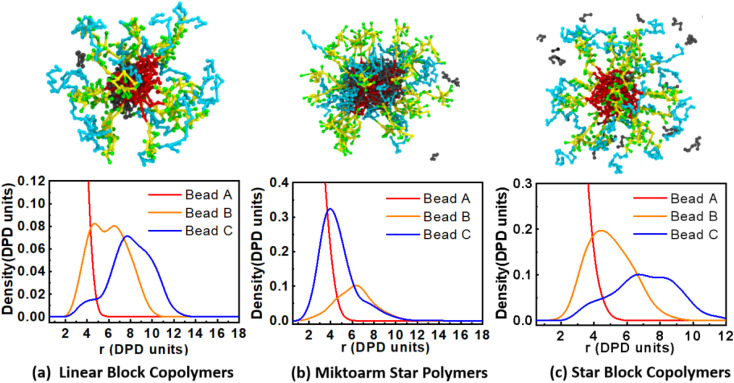
The density profiles for three spherical micelles at the degree of protonation *α*_H^+^_ = 100%: (a) linear block copolymers; (b) miktoarm star polymers; (c) star block copolymers.

The disintegrated states at full protonation (*α*_H^+^_ = 100%) in Fig. 8 are further characterized by the density profiles presented in Fig. 9. For the linear and star block copolymer micelles (Fig. 9a and c), the small aggregates exhibit a core–shell–corona architecture. In this configuration, the hydrophobic segments A form the core, the pH-sensitive segments B constitute the shell layer, and the hydrophilic segments C extend outward to form the corona. For the miktoarm star polymers (Fig. 9b), the hydrophobic segments A form the core, and the pH-sensitive segments B and the hydrophilic segments C collectively constitute a mixed shell layer.

To further investigate the topological effects on the drug release mechanisms, the mean square displacements (MSD) of beads Y_1_ have been calculated, which can help analyze the diffusion behavior of the drug molecules. MSD is defined as the ensemble average of squared displacements from initial positions over a time interval:^40^14
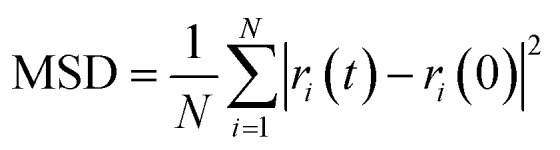


**Fig. 10:**
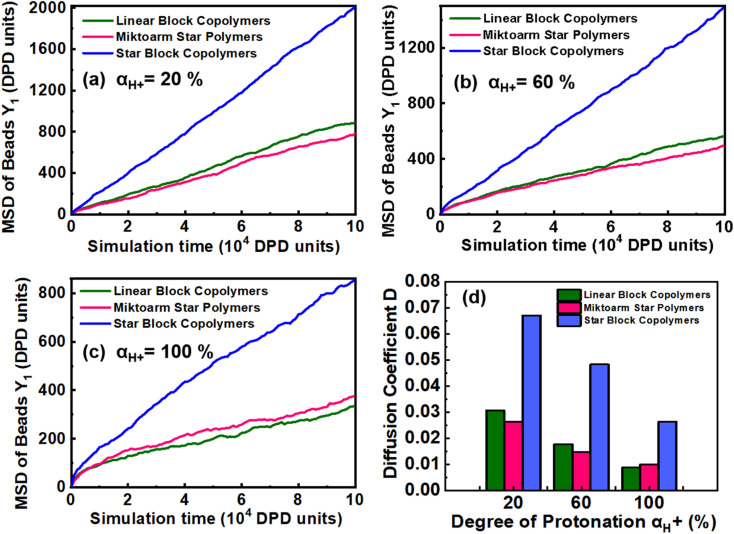
(a)–(c) Mean square displacement (MSD) of drug beads (Y_1_) over time for the three drug-loaded micelles at different degrees of protonation (*α*_H^+^_ = 20%, 60%, 100%). (d) Diffusion coefficient *D* of the drug at different protonation degrees. The results show that drug diffusion from linear (green) and block-star (blue) polymers is generally faster than from the miktoarm star polymer (pink).

The MSD of the drug molecules and their corresponding diffusion coefficients^40^ (*D*) are presented in Fig. 10. The MSD curves for the linear block copolymers and miktoarm star polymers display a gradual increase. This behavior indicates a sustained and controlled release pattern, which is a highly desirable characteristic for therapeutic applications requiring prolonged maintenance of effective drug concentrations.^11^ In contrast, drug molecules released from micelles formed by star block copolymers consistently demonstrate significantly higher MSD values and diffusion coefficients compared to those from linear block copolymers and miktoarm star polymers. The faster drug release has been observed in the system of star block copolymers: when the B-block shell swells due to protonation, the encapsulated drug molecules can release into solution rapidly. However, the drug release for the linear block copolymers is obviously slower, despite their identical ‘shell-loading’ mode.

**Fig. 11:**
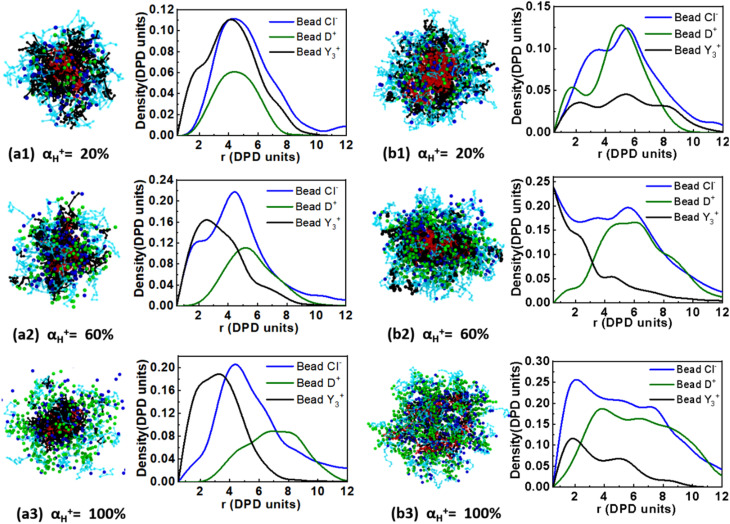
The morphologies of polymeric micelles at timestep = 1000 and density profiles of charged beads: (a1)–(a3) linear block copolymers; (b1)–(b3) star block copolymers. Bead representations: CI^−^ (blue), D^+^ (green), and Y_3_^+^ (black).

Notably, despite sharing the same “shell-loading” mode, micelles formed by the linear and star block copolymers exhibit marked discrepancies in drug release behaviors, as shown in Fig. 10. To investigate this phenomenon, we analyzed the ionic distribution at the micelle–solution interface at the initial stage (timestep = 1000, Fig. 11). For the linear block copolymeric micelles (Fig. 11a1–a3), Cl^−^ density in the hydrophilic layer consistently surpasses that of D^+^ under all simulated acidic conditions. This localized Cl^−^ enrichment generates a net negative electrostatic potential gradient, forming a substantial energy barrier that electrostatically impedes the outward diffusion of the cationic drug Y^3+^. The consistently observed higher Cl^−^ density relative to D^+^ in the linear micelle corona indicates an overcharging effect, creating a net negative electrostatic barrier that significantly restricts Y^3+^ diffusion. In contrast, the star block copolymeric micelles (Fig. 11b1–b3) exhibit a near-equilibrium density between Cl^−^ and D^+^ in their corona. This charge-balanced microenvironment generates a significantly weaker net electrostatic field, effectively neutralizing most electrostatic interactions. This fundamental difference in the interfacial electrostatic environment is directly attributable to the topological variations between the two polymer architectures. The linear block copolymers possess greater conformational freedom, enabling pH-responsive segments to migrate more rapidly to the hydrophilic layer during acidification. This rapid migration promotes localized Cl^−^ accumulation. Conversely, the mobility of pH-responsive segments in the star block copolymers is severely constrained by their central core tethering. This restricted motion results in slower, more gradual corona reorganization in response to pH changes, ultimately yielding a more balanced ion distribution rather than pronounced counterion enrichment.

## 4. Conclusion

This study systematically investigates the profound impact of topological architecture on the structure–property relationships of ABC-type polymer carriers through DPD simulations. Our findings demonstrate that, despite identical block compositions, three distinct topologies—linear block copolymers, miktoarm star polymers, and star block copolymers—exhibit fundamentally different drug distribution patterns and release mechanisms. Notably, the miktoarm star polymer uniquely enables a “core–shell dual loading” mode, wherein DOX molecules are simultaneously encapsulated in both the hydrophobic core and the pH-responsive intermediate layer. In contrast, linear and star block copolymers primarily demonstrate a “shell-loading” mode, where drugs are confined to the intermediate layer. Compared to the linear and star block copolymers, the miktoarm star polymers effectively balance drug-loading capacity and micellar stability. The effect of polymer topology on drug release mechanisms becomes even more evident. All systems undergo a pH-triggered structural transition to “firework-like” aggregates, while their release mechanisms diverge significantly. The micelles formed by the star block copolymers exhibit a faster drug release, while there occurs a gradual diffusion of drug molecules from the linear and miktoarm micelles. Notably, despite sharing the same “shell-loading” mode, micelles formed by linear and star block copolymers exhibit significant differences in drug release behavior. This divergence primarily stems from variations in the interfacial electrostatic environment caused by differences in topological structure: the conformational freedom of the linear structure leads to an overcharging effect, creating an electrostatic barrier, while the restricted motion of the star structure promotes a more balanced ion distribution. This research ultimately underscores that topological design provides an effective strategy for precisely tailoring both the spatial distribution and release kinetics of drugs within nanocarriers.

## Author contributions

Zengwei Ma: conceptualization, methodology, formal analysis, writing-original draft, funding acquisition. Gaiqin Liu: investigation, data curation, writing-review and editing. Jianwei Wei: project administration, supervision, visualization, writing-review and editing.

## Conflicts of interest

There are no conflicts to declare.

## Data Availability

The code for DL_MESO package can be found at https://www.ccp5.ac.uk/DL_MESO/. The version of the code employed for this study is version 2.7.

## References

[cit1] Liu M., Blankenship J. R., Levi A. E., Fu Q., Hudson Z. M., Bates C. M. (2022). Chem. Mater..

[cit2] Kuperkar K., Patel D., Atanase L. I., Bahadur P. (2022). Polymers.

[cit3] El Yousfi R., Brahmi M., Dalli M., Achalhi N., Azougagh O., Tahani A., Touzani R., El Idrissi A. (2023). Polymers.

[cit4] Wu W., Wang W., Li J. (2015). Prog. Polym. Sci..

[cit5] Aghajanzadeh M., Zamani M., Rostamizadeh K., Sharafi A., Danafar H. (2018). J. Macromol. Sci. Part A Pure Appl. Chem..

[cit6] Zhao Y. (2019). Macromol. Rapid Commun..

[cit7] Chen J., Qiu X., Ouyang J., Kong J., Zhong W., Xing M. M. Q. (2011). Biomacromolecules.

[cit8] Chen D., Song P., Jiang F., Meng X., Sui W., Shu C., Wan L.-J. (2013). J. Phys. Chem. B.

[cit9] Kocak G., Tuncer C., Bütün V. (2017). Polym. Chem..

[cit10] Shahriari M., Torchilin V. P., Taghdisi S. M., Abnous K., Ramezani M., Alibolandi M. (2020). Biomater. Sci..

[cit11] Luo L., Xu F., Peng H., Luo Y., Tian X., Battaglia G., Zhang H., Gong Q., Gu Z., Luo K. (2020). J. Controlled Release.

[cit12] Ding H., Tan P., Fu S., Tian X., Zhang H., Ma X., Gu Z., Luo K. (2022). J. Controlled Release.

[cit13] Fan S.-Y., Hao Y.-N., Zhang W.-X., Kapasi A., Shu Y., Wang J.-H., Chen W. (2020). ACS Appl. Mater. Interfaces.

[cit14] Yang Y. Q., Zhao B., Li Z. D., Lin W. J., Zhang C. Y., Guo X. D., Wang J. F., Zhang L. J. (2013). Acta Biomater..

[cit15] Zhou P., Liu Y.-Y., Niu L.-Y., Zhu J. (2015). Polym. Chem..

[cit16] Lotocki V., Kakkar A. (2020). Pharmaceutics.

[cit17] Baghbanbashi M., Kakkar A. (2022). Mol. Pharm..

[cit18] Kakkar A. (2025). Nanomed..

[cit19] Zhang X., Dai Y., Dai G., Deng C. (2020). RSC Adv..

[cit20] Karatzas A., Haataja J. S., Skoulas D., Bilalis P., Varlas S., Apostolidi P., Sofianopoulou S., Stratikos E., Houbenov N., Ikkala O., Iatrou H. (2019). Biomacromolecules.

[cit21] Datta S., Huntošová V., Jutková A., Seliga R., Kronek J., Tomkova A., Lenkavská L., Máčajová M., Bilčík B., Kundeková B., Čavarga I., Pavlova E., Šlouf M., Miškovský P., Jancura D. (2022). Pharmaceutics.

[cit22] Niesyto K., Keihankhadiv S., Mazur A., Mielańczyk A., Neugebauer D. (2024). Int. J. Mol. Sci..

[cit23] Datta S., Kronek J., Nadova Z., Timulakova L., Minarcikova A., Miskovsky P. (2025). Eur. J. Pharm. Biopharm..

[cit24] Dai Y., Wu D., Lin S., Ma X., Zhang X., Xia F. (2018). J. Nanopart. Res..

[cit25] Yuan Y.-Y., Wang J. (2011). Colloids Surf., B.

[cit26] Thota N., Jiang J. (2015). Front. Mater..

[cit27] Ramezani M., Shamsara J. (2016). J. Mol. Graphics Modell..

[cit28] Feng Y. H., Zhang X. P., Zhao Z. Q., Guo X. D. (2020). Mol. Pharm..

[cit29] Guo W. X., Hu L. F., Feng Y. H., Chen B. Z., Guo X. D. (2022). Colloids Surf., B.

[cit30] T A., Narayan R., Shenoy P. A., Nayak U. Y. (2022). J. Mol. Liq..

[cit31] Espanol P., Warren P. (1995). Europhys. Lett..

[cit32] Groot R. D., Warren P. B. (1997). J. Phys. Chem..

[cit33] González-Melchor M., Mayoral E., Velázquez M. E., Alejandre J. (2006). J. Phys. Chem..

[cit34] Wang J., Han Y., Xu Z., Yang X., Ramakrishna S., Liu Y. (2021). Macromol. Mater. Eng..

[cit35] Gao P., Jiang X., Li J., Nicolas J., Ha-Duong T. (2026). Adv. Healthcare Mater..

[cit36] Luo Z., Jiang J. (2012). J. Controlled Release.

[cit37] Nie S. Y., Lin W. J., Yao N., Guo X. D., Zhang L. J. (2014). ACS Appl. Mater. Interfaces.

[cit38] Lin W. J., Nie S. Y., Chen Q., Qian Y., Wen X. F., Zhang L. J. (2014). AIChE J..

[cit39] Yang Z., Mai H., Wang D., He T., Chen F., Yang C. (2023). ACS Omega.

[cit40] Wu W., Yi P., Zhang J., Cheng Y., Li Z., Hao X., Chen Q. (2019). Phys. Chem. Chem. Phys..

[cit41] Hoogerbrugge P. J., Koelman J. (1992). Europhys. Lett..

[cit42] Procházka K., Limpouchová Z., Štěpánek M., Šindelka K., Lísal M. (2022). Polymers.

[cit43] Min W., Zhao D., Quan X., Sun D., Li L., Zhou J. (2017). Colloids Surf., B.

[cit44] Guo X. D., Qian Y., Zhang C. Y., Nie S. Y., Zhang L. J. (2012). Soft Mater..

[cit45] Posel Z., Limpouchová Z., Šindelka K., Lísal M., Procházka K. (2014). Macromolecules.

[cit46] Mai J., Sun D., Li L., Zhou J. (2016). J. Chem. Eng. Data.

[cit47] Seaton M. A., Anderson R. L., Metz S., Smith W. (2013). Mol. Simul..

[cit48] Humphrey W., Dalke A., Schulten K. (1996). J. Mol. Graph..

[cit49] Ren J. M., McKenzie T. G., Fu Q., Wong E. H. H., Xu J., An Z., Shanmugam S., Davis T. P., Boyer C., Qiao G. G. (2016). Chem. Rev..

[cit50] Lin W., Nie S., Zhong Q., Yang Y., Cai C., Wang J., Zhang L. (2014). J. Mater. Chem. B.

[cit51] Wen X.-f., Lan J.-l., Cai Z.-q., Pi P.-h., Xu S.-p., Zhang L.-j., Qian Y., Wang S.-n. (2014). J. Nanopart. Res..

[cit52] Prhashanna A., Tan W. K., Khan S. A., Chen S. B. (2016). Colloids Surf., B.

[cit53] Zeng S., Quan X., Zhu H., Sun D., Miao Z., Zhang L., Zhou J. (2021). Langmuir.

[cit54] Zhang C., Liu T., Wang W., Bell C. A., Han Y., Fu C., Peng H., Tan X., Král P., Gaus K., Gooding J. J., Whittaker A. K. (2020). ACS Nano.

